# Moderate-Vigorous Physical Activity across Body Mass Index in Females: Moderating Effect of Endocannabinoids and Temperament

**DOI:** 10.1371/journal.pone.0104534

**Published:** 2014-08-07

**Authors:** Fernando Fernández-Aranda, Sarah Sauchelli, Antoni Pastor, Marcela L. Gonzalez, Rafael de la Torre, Roser Granero, Susana Jiménez-Murcia, Rosa Baños, Cristina Botella, Jose M. Fernández-Real, Jose C. Fernández-García, Gema Frühbeck, Javier Gómez-Ambrosi, Roser Rodríguez, Francisco J. Tinahones, Jon Arcelus, Ana B. Fagundo, Zaida Agüera, Jordi Miró, Felipe F. Casanueva

**Affiliations:** 1 Department of Psychiatry, University Hospital of Bellvitge-IDIBELL, Barcelona, Spain; 2 CIBER Fisiopatología Obesidad y Nutrición (CIBERobn), Instituto Salud Carlos III, Madrid, Spain; 3 Department of Clinical Sciences, School of Medicine, University of Barcelona, Barcelona, Spain; 4 Department of Pharmacology, School of Medicine, Universitat Autònoma de Barcelona, Barcelona, Spain; 5 Human Pharmacology and Clinical Neurosciences Research Group, Neuroscience Research Program, IMIM (Hospital del Mar Medical Research Institute), Barcelona, Spain; 6 Department of Psychology, Universitat Rovira i Virgili, Tarragona, Spain; 7 Department of Experimental and Health Sciences, Universitat Pompeu Fabra, Barcelona, Spain; 8 Departament de Psicobiologia i Metodologia, Universitat Autònoma de Barcelona, Barcelona, Spain; 9 Department of Psychological, Personality, Evaluation and Treatment of the University of Valencia, Valencia, Spain; 10 Department of Basic Psychology, Clinic and Psychobiology of the University Jaume I, Castelló, Spain; 11 Department of Diabetes, Endocrinology and Nutrition, Institut d’Investigació Biomèdica de Girona (IdlBGi) Hospital Dr Josep Trueta, Girona, Spain; 12 Department of Diabetes, Endocrinology and Nutrition, Hospital Clínico Universitario Virgen de Victoria, Málaga, Spain; 13 Department of Endocrinology and Nutrition, Clínica Universidad de Navarra, University of Navarra, Pamplona, Spain; 14 Eating Disorders Service, Glenfield University Hospital, Leicester, United Kingdom; 15 Department of Medicine, Endocrinology Division, Santiago de Compostela University, Complejo Hospitalario Universitario, Santiago de Compostela, Spain; Complexo Hospitalario Universitario de Santiago, Spain

## Abstract

**Background:**

Endocannabinoids and temperament traits have been linked to both physical activity and body mass index (BMI) however no study has explored how these factors interact in females. The aims of this cross-sectional study were to 1) examine differences among distinct BMI groups on daytime physical activity and time spent in moderate-vigorous physical activity (MVPA), temperament traits and plasma endocannabinoid concentrations; and 2) explore the association and interaction between MVPA, temperament, endocannabinoids and BMI.

**Methods:**

Physical activity was measured with the wrist-worn accelerometer Actiwatch AW7, in a sample of 189 female participants (43 morbid obese, 30 obese, and 116 healthy-weight controls). The Temperament and Character Inventory-Revised questionnaire was used to assess personality traits. BMI was calculated by bioelectrical impedance analysis via the TANITA digital scale. Blood analyses were conducted to measure levels of endocannabinoids and endocannabinoid-related compounds. Path-analysis was performed to examine the association between predictive variables and MVPA.

**Results:**

Obese groups showed lower MVPA and dysfunctional temperament traits compared to healthy-weight controls. Plasma concentrations of 2-arachidonoylglyceryl (2-AG) were greater in obese groups. Path-analysis identified a direct effect between greater MVPA and low BMI (*b* = −0.13, *p* = .039) and high MVPA levels were associated with elevated anandamide (AEA) levels (*b* = 0.16, *p* = .049) and *N*-oleylethanolamide (OEA) levels (*b* = 0.22, *p* = .004), as well as high Novelty seeking (*b* = 0.18, *p*<.001) and low Harm avoidance (*b* = −0.16, *p*<.001).

**Conclusions:**

Obese individuals showed a distinct temperament profile and circulating endocannabinoids compared to controls. Temperament and endocannabinoids may act as moderators of the low MVPA in obesity.

## Introduction

There is a growing prevalence of obesity and overweight worldwide. A fall in energy expenditure is believed to be one of the leading lifestyle changes boosting the notable spread of obesity [1]. Prolonged sedentary behavior has been strongly associated with this extreme weight condition and a consequent increase in the likelihood of cardiovascular diseases, hypertension, type 2 diabetes, and osteoporosis [2]. Differently, regular exercise or structured moderate-vigorous physical activity (MVPA) facilitates weight control [3].

These changes in physical activity (PA) patterns are of particular concern for females. The prevalence of obesity has been estimated to be greater among females than males (11.9% versus 7.7%) across all studied world regions [2]. In addition, females spend less time engaging in MVPA and more in sedentary activities, a gender difference especially seen among younger adults [4]. Given this evidence, and that the decline in PA over time is especially present among females [5], there is an urgent need to understand the mechanisms underlying the inter-individual fluctuations in structured PA and their relationship to body mass index (BMI) in females.

### Moderate-Vigorous Physical Activity /BMI and Endocannabinoids

Biological models have been proposed to explain individual differences in MVPA. Studies have identified the role of the endocannabinoid (eCB) system in the engagement and maintenance of structured PA, interacting with the reward neurosystem of exercise [6], [7]. It has been suggested that the beneficial effects of PA for cognitive function may be partly related to the eCB system [8]. The eCB system is also known for its modulation of cognitive and emotional behavior [9] and for its extensive central and peripheral control of energy balance [10]. The eCBs 2-arachidonoylglycerol (2-AG) and anandamide (AEA) are the endogenous lipid mediators of this system and have similar actions to those of the exogenous plant cannabinoid Δ9-tetrahydrocannabionol (THC). ECBs stimulate appetite and food intake by intensifying the orosensory reward of food, which takes place via the activation of the CB1 receptors of the central nervous system. It is also believed that the motivation to ingest is modulated by interactions between the eCB and opioid systems [11]. ECBs may additionally be involved in the peripheral regulation of feeding since intestinal levels of AEA have been found to increase under food deprivation and decrease during re-feeding [12]. The opposite pattern occurs with N-oleoylethanolamide (OEA), an eCB-related compound but non-CB1 receptor ligand with anorectic effects [13]. Upon the ingestion of fat, OEA is formed in the intestine and activates the intestinal peroxisome proliferator-activated receptor alpha (PPARα), which sends a satiety signal through the vagus nerve [14].

An over-activation of the eCB system has been associated with obesity and abnormal eating behavior [15]. The eCB system is also involved in lipid and glucose metabolism and its peripheral dysregulation in obesity affects several organs that participate in energy homeostasis including the liver, pancreas, adipose tissue and skeletal muscle [16]. Studies of the eCB system in human subjects have reported that in the obese condition plasma levels of 2-AG are increased [17], [18], while other studies have also reported elevated AEA plasma levels in obese subjects compared to lean subjects [17].

In animal and human studies [11], [19], [20], both N-acylethanolamides AEA and OEA, as well as other analogs such as N-palmitoylethanolamide (PEA), have been found to increase shortly after intense exercise, while 2-AG seems to remain stable. You et al [21] found that the gene expression of fatty acid amide hydrolase (FAAH), enzyme that degrades the N-acyl-ethanolamides, is lower in the abdominal adipose of obese women on a program combining exercise training and a caloric restriction diet. Dubreucq et al [22] reported CB1 knockout mice to display 30–40% less running behavior, and proposed a functional loop between the eCB system and MVPA. In support, studies have found that acute administrations of CB1 receptor antagonists or knocking down CB1 receptors in brain GABA neurons have a negative effect on wheel running activity in rats and mice [23]. Further, Avraham et al. [24] administered the eCBs-related compound 2-arachidonylglyceryl-ether (2-AGE, Noladin) in Sabra mice, detecting that high doses of 2-AGE did not alter food intake, but resulted in weight loss and increased PA. These findings may suggest an interaction between eCBs, BMI, MVPA and reward circuits.

### Moderate-Vigorous Physical Activity/BMI and Temperament

Cloninger [25] proposed a psychobiological model of personality comprising four heritable temperament traits: Novelty seeking, Harm avoidance, Reward dependence, and Persistence. Some of Cloninger’s temperament traits have been associated with MVPA. Authors found a negative correlation between MVPA and Harm avoidance (characterized by inhibition, anxiety and a pessimistic attitude) and a positive link to low Novelty seeking (characterized by introversion, lack of enthusiasm, tolerance to monotony, low response to novelty and low dynamism and curiosity) [26]. In a meta-analysis, the personality trait extraversion, conceptually related to Novelty seeking, and conscientiousness appeared to have a positive effect on structured PA, while high scores of neuroticism, opposite to extraversion, to be inversely associated with MVPA [27]. A relationship between specific temperament traits and BMI has also been described in the literature. Several studies have connected Novelty seeking with obesity, although some observe a positive association [28], while others did not find significant differences [29]. Further, greater Harm avoidance scores were found in obese compared to lean participants [30].

### Endocannabinoids and Temperament

The eCB system plays a modulator role in many cognitive and emotional processes [9]. Studies have shown a link between the eCB system and Cloninger’s temperament traits. An inverse relationship was observed between CB1 receptor availability and Novelty seeking [31]. Further, AEA has been identified as a substrate of the cytochrome P450 2D6 [32], and genotypic variations of this enzyme have been linked to individual differences in Harm avoidance, socialization ability, and anxiety [33]. Navarrete et al [34] compared the genetic expression of dopamine (DRD2) and cannabinoid (CB1, CB2) receptors in two mouse strains, observing that the mouse strain displaying greater motor behavior also presented more exploratory behavior, impulsivity, and lower attention capacity, and that these were related to CB2 receptor regulation.

### Aims of the study

The literature demonstrates significant relationships between the eCB system, Cloninger’s temperament traits and MVPA, all associated with BMI. However, no study has analyzed these factors together to assess the modulating effect of eCB functioning and temperament traits on MVPA and lifestyle PA, and the links to BMI in females. Therefore, the aims of the present study were to: 1) examine differences among distinct BMI groups on lifestyle PA levels and time spent in MVPA, temperament traits and plasma eCB concentrations; and 2) explore the association and interaction between MVPA, temperament, eCBs and BMI. Based on the literature, we hypothesized that: 1) Higher BMI would be associated with greater sedentary behavior, altered plasma eCBs levels and a specific temperament profile; 2) MVPA levels would be linked to specific temperament traits, in particular Novelty seeking, and altered eCB concentration, in particular augmented plasma AEA levels; 3) Both temperament and eCBs would be implicated in the relationship between MVPA and BMI.

## Materials and Methods

### Ethics statement

All participants gave written informed consent and the Ethics Committees of all the research institutions involved in the data collection approved the study: Comité Ético de Investigación Clínica del Hospital Universitari de Bellvitge; Comitè Ètic d’Investigació Clínica del Hospital Universitari de Girona Doctor Josep Trueta; Comité Ético de Investigación Clínica del Consorci Mar Parc de Salut de Barcelona-Parc de Salut Mar; Subcomisión de Investigación Clínica del Hospital Universitario “Virgen de la Victoria”; Comité Ético de Investigación Clínica de la Universidad de Navarra; Comité Ético de Investigación Clínica de Galicia & Universidad de Santiago de Campostela; Comissió Deontológica de la Universitat Jaume 1. The study was conducted in accordance with the Declaration of Helsinki.

### Participants

The sample comprised 189 female individuals, distributed along the BMI continuum, and included: 30 obese participants (BMI = 30–39.9, kg/m^2^), 43 morbid obese (BMI ≥ 40, kg/m^2^), and 116 healthy-weight controls (BMI = 18.5–29.9 kg/m^2^). Participants were Spanish speakers, with a mean age of 34 years (SD = 12.3) (distribution of mean age by group was: control 27.6 –SD = 7.9–, obese 44.9 –SD = 12.9– and morbid obese 43.5 –SD = 10.2–). Seven centers, all involved in the CIBERobn Spanish Research Network, participated: the Eating Disorders Unit (Department of Psychiatry, University Hospital of Bellvitge-IDIBELL, Barcelona), the Department of Endocrinology at the University Hospital of Santiago (Santiago de Compostela); the Department of Diabetes, Endocrinology and Nutrition (Clinic University Hospital Virgen de Victoria, Malaga); the Department of Endocrinology and Nutrition (University of Navarra, Pamplona); the Diabetes, Endocrinology and Nutrition Department, Biomedical Research Institute of Girona (IdIBGi-Doctor Josep Trueta Hospital, Girona); the Hospital del Mar Medical Research Institute (IMIM, Barcelona) and the Department of Basic Psychology, Clinic and Psychobiology (University Jaume I, Castellón). The obese participants were patients who had been consecutively referred to the clinics mentioned above. Recruitment of the controls took place by means of word-of-mouth and advertisements at the local universities. All controls were from the same catchment area as the obese patients.

Exclusion criteria were: a) having suffered a lifetime history of Axis I mental disorders since many are linked to altered PA and eCB levels and temperament styles, especially depression and eating disorders, which are highly co-morbid with obesity [35], [37]; b) having a history of chronic medical illness or a neurological condition (e.g. Parkinson’s disease) that may affect motor capacity; c) use psychoactive medication or drugs that influence PA (e.g. cocaine, beta blockers or thyroid medication) or plasma endocannabinoid concentrations (e.g. cannabis); d) being under 18, as adolescence is characterized by psychobiological changes, or over 60 given that age-related medical conditions (e.g. arthritis) affect physical functioning in daily life. Substance abuse/dependence (including cannabis) and eating disorder diagnoses were conducted face-to-face using the Structured Clinical Interview for DSM-IV Axis I Disorders (SCID-I) [38]. The evaluation of general health or mental illnesses was based on the General Health Questionnaire-28 (GHQ-28) [39]. Enrolment into the study was between January 2010 and March 2013.

### Measures

#### Temperament and Character Inventory-Revised (TCI-R) [40]

This questionnaire is composed of 240-items scored on a 5-point Likert scale and measures personality derived from three character and four temperament dimensions. The dimensions reflecting temperament (Harm Avoidance, Novelty seeking, Reward Dependence and Persistence) were assessed, which entailed the analysis of 133 items of the total items in the questionnaire. Evaluation of the Spanish revised version [41] generated an internal consistency (coefficient alpha) of 0.87.

**Physical Activity** was evaluated with Actiwatch AW7 (Actiwatch AW7; CamNtech Ltd, Cambridge Neurotechnology, Cambridge, UK), a small (39×32×9 mm), light-weight (10.5 g) accelerometer that measures activity. The Actiwatch is worn on the non-dominant wrist for 6 days (4 week days and 1 weekend), from 00∶00 hr on day 1 to 00∶00 hr on day 7. PA data was calculated in the form of activity counts in a 1-minute epoch length over 24 hours. The counts represent the peak intensity of the movement detected by the Actiwatch AW7. Only the data between 7∶00hr and 23∶00hr was analyzed; a data reduction procedure that has been recommended and conducted in previous studies [42], [43]. No detected movement for 10 or more consecutive epochs (10 minutes) was considered as missing (seen as implausible counts or as periods in which the participant was sleeping). In addition, a minimum of 4 days of wear was used as criterion to accept the case. This is the lower recommended minimum to accurately estimate daily PA in adults [42]. Upon analysis of the data, there were no cases of 4 or less days of wear. The Actiwatch 7 software (CamNtech Ltd) was used to extract the data. Two PA variables were assessed:

#### Daytime PA Daily

PA was calculated in the form of mean counts per minute (counts·min^−1^) over the 6 days.

#### Time in SLPA and MVPA

The average amount of time during the day spent in sedentary-light PA (SLPA) and MVPA was calculated using an algorithm proposed by Heil [44]. Employing the activity monitor Actical (Mini Mitter Co., Inc., Bend, OR), another Actiwatch produced by the same manufacturer, which was placed on the ankle, hip and wrist, Heil [44] developed algorithms to predict activity energy expenditure (AEE) in children and adults. To obtain the cut point for MVPA, the formula: *AEE = 0.02013+(1.282E-5) x HAC* (elaborated for wrist worn accelerometers) was used. This yielded a cut point of 848 counts·min^−1^. This value predicts a PA intensity of 3 MET, which corresponds to a brisk walk. The algorithm to predict AEE in children has been used in a previous study to identify MVPA from the wrist-worn Actiwatch AW4 (CamNtech Ltd, Cambridge Neurotechnology, Cambridge, UK), an earlier version of the Actiwatch AW7 [45].

The Actiwatch AW4 has reliability as a measure of PA similar to other accelerometers [46]. Wrist worn accelerometers have been used to measure PA in various studies [45], [47]. They have also been found to predict a similar amount of variance in energy expenditure to the hip-placed accelerometers [48], [49].

**Body Composition** was assessed using the Tanita Multi-Frequency Body Composition Analyzer MC-180MA (Tanita Corporation, Tokyo, Japan). The Tanita is a weighting instrument utilizing bioelectrical impedance analysis for the screening of body fat and composition. This instrument is repeatedly revised in relation to the reference standards dual-energy X-ray absorptiometry (DEXA) (http://www.bl-biologica.es/tanita_tbf.htm) and has been validated against other weighing methods [50]. Height was calculated using a stadiometer.

#### Endocannabinoids quantification method

Blood samples were collected from participants between 8 and 9 am after at least 12 hours of fasting. The blood was centrifuged at 3500 rpm at 4°C for 15–20 min. Plasma aliquots were stored at −80°C until analysis. Plasma concentrations of the eCBs AEA (ng/mL) and 2-AG (ng/mL) were assessed. In addition, the following acylethanolamides OEA (ng/mL) and PEA (ng/mL) were assessed.

The eCB quantification was done with modifications of a previously described methodology of eCB analysis in brain tissue [51]. After adding the following amounts of deuterated analogues (Cayman Chemical, USA) 0.25 ng AEA-d4, 1 ng PEA-d4 and OEA-d4, 5 ng 2-AG to a 0.5 mL aliquot of plasma, eCBs were extracted with a liquid-liquid extraction in tert-butyl-methyl-ether (Merck, Germany) and the extracts analyzed in a LC/MS-MS system (Agilent 6410, USA). ECBs were separated in a C8 column (2.1×100 mm×1.8 µm particle size, Zorbax, Agilent) by gradient chromatography of a mobile phase of water and acetonitrile containing 0.1% formic acid (Merck, Germany). The source operated on the positive electrospray ionization mode and the detection was done by the multiple reactions monitoring mechanism (MRM). The following precursor to product ion transitions were used: m/z 379→287 for 2-AG, m/z 348→62 for AEA, m/z 326→62 for OEA, m/z 300→62 for PEA, m/z 384→287 for 2-AG-d5, 352→66 for AEA-d4, m/z 330→66 for OEA-d4 and m/z 304→66 for PEA-d4. ECB quantification was done by isotopic dilution of the deuterated analogues response. Variations in precision and accuracy were<15% for the individual sample replicates.

### Procedure

Experienced psychologists and psychiatrists (all extensively trained in the use of the instruments) completed the clinical and physical assessment in two structured face-to-face interviews. In addition to the first clinical interview, temperament and general health status information was obtained through self-report questionnaires. Prior to assessment, basic anthropometrical features were determined by the TANITA and blood samples were obtained after overnight fasting. The accelerometers provided in the first interview were collected after 7 days in a second face-to-face assessment session.

### Statistical Analysis

Statistical analysis was carried out with STATA13 for Windows. Analysis of variance (ANOVA) was used to compare BMI, PA level and eCBs between diagnostic subtypes (controls, obese and morbid obese). Polynomial contrasts into ANOVA were explored by means of linear and quadratic trends, and post-hoc comparisons and Cohens’-d coefficients for the effect size of differences between groups (moderate effect size was considered for |*d*|≥0.50 and good effect size for |*d*|≥0.80).

Structural equation models (SEM) tested the mediational pathway between temperament scores, MVPA levels, eCBs and BMI, adjusted by the covariate participants’ age. The mediational path was considered as adequate when it met previously described criteria [52]. Overall goodness-of-fit statistics were assessed with the χ^2^ test, the root mean squared error of approximation (RMSEA), baseline comparison indexes (Comparative Fit Index CFI and Tucker-Lewis Index TLI) and residuals size (Standardized Mean Squared Residual SMSR). A fit was considered to be good if [53]: a non-significant result (*p*>.05) was achieved for the χ^2^ test, the RMSEA was<.08, the CFI-TI coefficients were>.90 and SRMR was limited to 0.08. The equation level goodness-of-fit and the effect sizes were estimated through multiple correlation (mc) and Bentler-Raykov multiple correlation (mc2) [54].

## Results

### Comparison of Physical Activity measures, BMI, Temperament and Endocannabinoids between groups

Results obtained in ANOVA procedures (Table 1) showed differences between groups of weight for the MVPA means: a negative linear trend emerged (the higher the weights the lower the MVPA mean levels, *p* = .008) and statistical differences for the pairwise comparison between morbid obese versus controls (f = −19.2, *p* = .005) were found. No statistical association emerged between groups of weight and daytime PA levels. Linear trends appeared for TCI-R temperament scales: Novelty seeking (decreasing trend), Harm avoidance (increasing trend) and Persistence scales (increasing trend), and an additional quadratic trend for Reward dependence. Statistical differences were found between obese and controls for Novelty seeking (*p* = .015), Harm avoidance (*p*<.001) and Reward dependence (*p* = .002), and between morbid obese and controls for Novelty seeking (*p* = .038) and Harm avoidance (*p*<.001). As shown in Table 2, the eCB 2-AG was associated with the distinct BMI groups: positive linear and quadratic trends were obtained for this biological measure and statistical differences emerged in the post-hoc comparison between both obese and morbid obese versus controls.

**Table 1:** BMI, PA, temperament, endocannabinoids and endocannabinoids related compounds among study groups.

	Controls		Obese		Morbid-obese		ANOVA: p-value, trends and contrasts								
	(*n* = 116)		(*n* = 30)		(*n* = 43)		Group	LT	QT	OB vs CO		MO vs CO		MO vs OB	
	Mean	SD	Mean	SD	Mean	SD	*p*	*p*	*p*	φ	| d |	φ	| d |	φ	| d |
Body mass index	21.6	2.8	35.5	2.3	46.2	4.8	**<.001**	**<.001**	**.019**	**13.9** *	*5.42*	24.6*	*6.24*	**10.7** *	*2.81*
MVPA	67.20	35.18	65.84	51.03	48.02	33.03	**.016**	**.008**	.283	**–**1.35	0.03	**–19.18** *	*0.56*	**–17.82** *	0.41
Daytime PA	297.97	68.77	305.31	93.16	273.71	72.02	.120	.109	.196	7.34	0.09	**–**24.26	0.34	**–**31.60	0.38
TCI-R: Novelty-seeking	100.3	12.9	92.9	18.1	94.8	14.1	**.017**	**.038**	.120	**–7.35** *	0.47	**–5.51** *	0.41	1.84	0.11
TCI-R: Harm avoidance	92.2	16.1	106.7	18.7	113.0	19.3	**<.001**	**<.001**	.261	**14.5** *	*0.83*	**20.8** *	*1.17*	6.29	0.33
TCI-R: Reward depend.	99.6	14.4	109.0	14.6	103.0	13.9	**.008**	.194	**.010**	**9.38** *	*0.65*	3.40	0.24	**–**5.98	0.42
TCI-R: Persistence	111.4	16.8	112.8	20.0	104.9	19.6	.100	**.050**	.210	1.44	0.08	**–**6.51	0.36	**–**7.95	0.40
2-AG (ng/mL)	1.60	1.02	3.34	1.65	3.82	2.55	**<.001**	**<.001**	.070	**1.74** *	*1.27*	**2.22** *	*1.14*	0.48	0.23
AEA (ng/mL)	0.55	0.17	0.59	0.26	0.62	0.20	.160	.069	.874	0.04	0.19	0.07	0.39	0.03	0.13
PEA (ng/mL)	2.48	0.61	2.62	0.94	2.41	0.50	.503	.603	.254	0.13	0.17	**–**0.07	0.12	**–**0.20	0.27
OEA (ng/mL)	4.07	1.39	4.13	1.58	3.86	0.89	.672	.425	.567	0.06	0.04	**–**0.22	0.19	**–**0.28	0.22

φ: Adjusted mean difference in ANOVA. LT: linear trend. QT: quadratic trend. | d |: Cohen’s-d.

*Bold: significant contrast (.05 level). Italics: moderate to good effect-size (| d |≥0.5).

BMI: Body mass index; MVPA: moderate-vigorous physical activity; PA: physical activity; 2-AG: 2- arachidonoylglycerol; AEA: anandamide; OEA: *N*-oleylethanolamide; PEA: *N*-palmitoylethanolamine.

**Table 2:** Results of the SEM evaluating the pathways between personality, MVPA, eCBs and BMI.

		Std.Coef.	SE	z	*p*	95% CI
MVPA	Novelty seeking	0.1761	0.0792	2.22	0.026	0.0208; 0.3314
	Harm avoidance	−0.1575	0.0795	−1.98	0.048	−0.3132; −0.0017
	*Constant*	*1.2444*	*0.7831*	*1.59*	*0.112*	−*0.2904;* *2.7792*
BMI	MVPA	−0.1285	0.0622	−2.06	0.039	−0.2505; −0.0065
	AEA	0.2649	0.0739	3.58	<0.001	0.1200; 0.4099
	OEA	−0.1514	0.0740	−2.05	0.041	−0.2964; −0.0065
	Novelty seeking	0.0317	0.0623	0.51	0.611	−0.0904; 0.1537
	Harm avoidance	0.2686	0.0608	4.42	<0.001	0.1494; 0.3879
	AGE (covariate)	0.5229	0.0551	9.49	<0.001	0.4149; 0.6309
	*Constant*	−*0.3051*	*0.6229*	−*0.49*	*0.624*	−*1.5260;* *0.9157*
AEA	MVPA	0.1558	0.0791	1.97	0.049	0.0007; 0.3109
	*Constant*	*2.7223*	*0.2386*	*11.41*	*<0.001*	*2.2546;* *3.1900*
OEA	MVPA	0.2233	0.0771	2.90	0.004	0.0722; 0.3743
	*Constant*	*2.6724*	*0.2423*	*11.03*	*<0.001*	*2.1974;* *3.1473*

### Mediation model of Moderate-Vigorous Physical Activity level when including temperament and biological parameters

**Figure 1:**
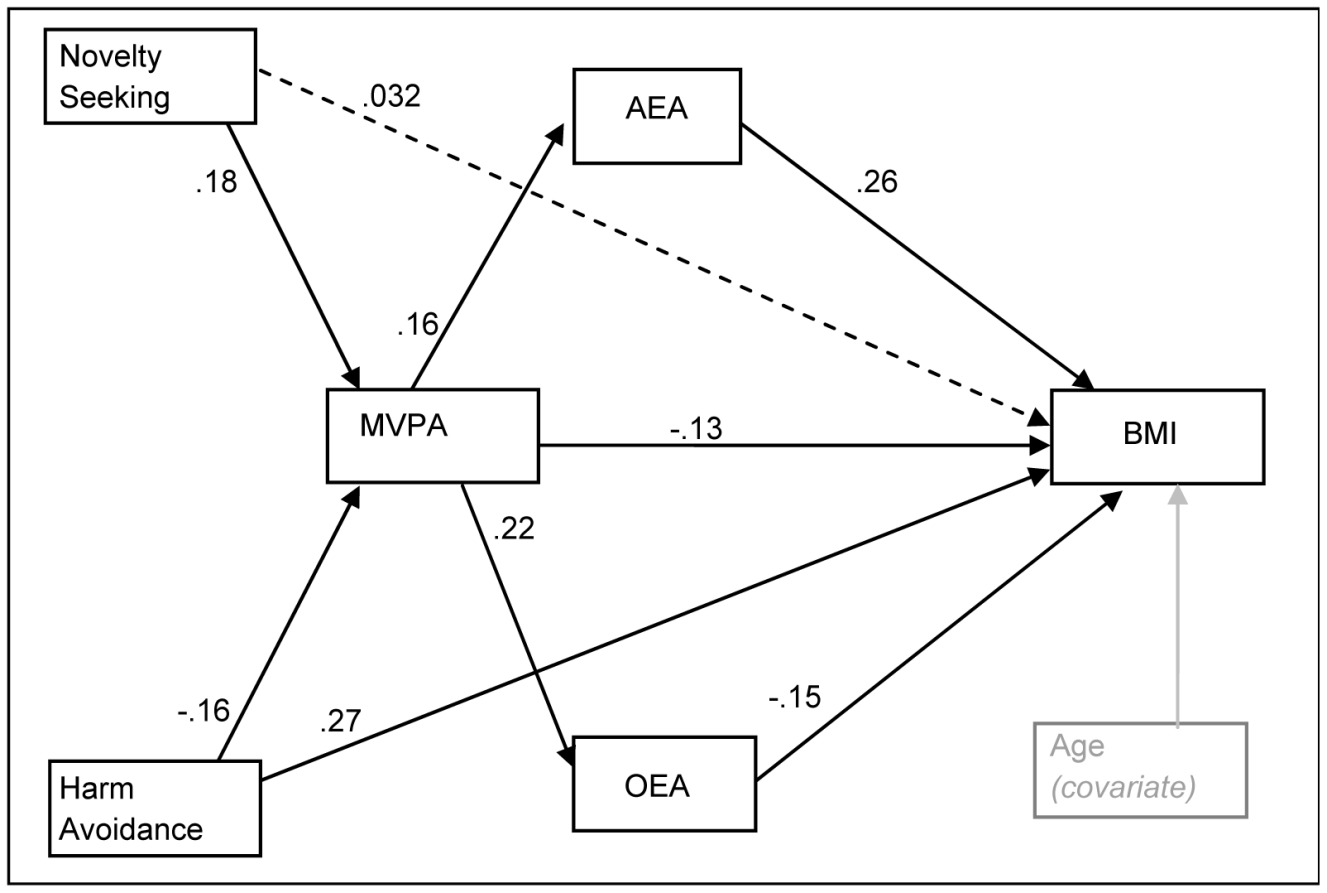
The moderating role of temperament and endocannabinoids on physical activity levels and body mass index. Continuous line: significant parameter. Structural Equation Model analysis shows that the temperaments low Novelty seeking and low Harm avoidance were predictive of low physical activity (MVPA) levels. High MVPA levels were associated with high anandamide (AEA) levels and high AEA levels were associated with high body mass index (BMI). In addition, high MVPA levels were associated with high *N*-oleylethanolamine (OEA) levels and low OEA levels were associated with high BMI. A direct effect was found between high MVPA and low BMI and between high Harm avoidance and high BMI.

Figure 1 shows the path-diagram and standardized structural coefficients for the mediational model between temperament traits, eCBs, MVPA level and BMI. Results were adjusted by the covariate participants’ age. Variables selected for the model accomplished Baron-Kenny’s requirements for mediational paths. No reciprocal association between eCBs and BMI were retained since no statistical effect of eCBs on BMI emerged, and retaining these parameters affected the fitting. Both low Novelty seeking and high Harm avoidance scores were predictive of lower MVPA levels. The eCBs measures AEA and OEA mediated the association between MVPA levels and BMI: a) high MVPA levels were associated with high AEA measures, and elevated AEA values were related to high BMI, b) high MVPA levels were also related to high OEA levels, and low levels for this cannabimimetic were associated with higher BMI. MVPA levels and Harm avoidance scores also showed direct effects with BMI (high BMI was predicted by high Harm avoidance and low MVPA levels). Pathway of Figure 1 achieved goodness-of-fit: χ^2^ = 8.36 (*p* = .30), RMSEA = .036, CFI = .99, TLI = .98 and SRMR = .033. Considering each equation level, MVPA level achieved low effect size values (mc = .26 and mc^2^ = .07), while BMI obtained higher ones (mc = .69 and mc^2^ = .48). The overall R^2^ (coefficient of determination) was very good (R^2^ = 0.47).

## Discussion

The aims of the present study were to analyze the differences between extreme BMI groups on daytime PA, MVPA, temperament and eCBs, and explore whether temperament traits and eCB concentrations are mediators of MVPA in females.

### Physical Activity and BMI

As expected, comparison among weight categories showed that the morbid obese participants displayed the least MVPA compared to the healthy-weight participants. Differently, daytime PA did not vary between groups. These findings are in line with the existing literature. A recent study assessing the effects of adherence to MVPA guidelines on BMI and waist circumference detected an inverse association between meeting the PA guidelines and baseline BMI and waist circumference. However, later linear regressions demonstrated that only vigorous PA was significantly correlated with lower BMI [55]. In addition, the authors observed that only high adherence to the MVPA guidelines resulted in decreases in BMI and waist circumference, while stable PA had no effect. Similarly, in another study, a significant trend across weight categories in PA among females was only observed for the time spent in vigorous PA [1]. A hypothesis may be that it is a gradual decrease in time spent in MVPA, rather than daytime activity per se that increases the risk and maintains the global prevalence of obesity. As the present study was a cross-sectional, causality cannot be determined, but our findings suggest that the relationship is more complex.

### Temperament and BMI

Regarding temperament, both obese groups (obese and morbid obese), presented a distinct temperament profile from the controls, characterized by greater scores in Harm avoidance and Reward dependence, but lower Novelty seeking. Whereas those studies that included obese participants with comorbid eating disorders (namely Binge Eating Disorder) found higher impulsivity scores [36], those who excluded this group of obese patients reported lower impulsivity levels [30]. This may explain the reason why the high Novelty seeking in obese individuals described in the literature [28] was not found in the present study, as individuals with a history of eating disorders were excluded from it. Similar to previous studies [30], the obese individuals in our study presented a temperament profile characterized by passivity, sensitivity, nervousness, insecurity, and social dependence.

### Endocannabinoid concentrations and BMI

One remarkable finding in our study was the elevated level of plasma 2-AG concentrations found in the obese groups, which is keeping with the literature [15]. Di Marzo et al [56] observed that a lifestyle modification program for obese patients resulted in a fall of both 2-AG and AEA concentrations, however only 2-AG was associated with a decrease in visceral adipose tissue, triacylglycerol concentrations, and HDL3-cholesterol concentrations, which suggests that these eCBs may have distinct metabolic roles. The present study therefore provides further support to the increasing evidence for the involvement of eCBs in fat metabolism development. Additional research must be conducted to determine the role of the eCB system in obesity.

### Temperament traits and endocannabinoid factors as mediators of Moderate-Vigorous Physical Activity and BMI

As expected, objectively measured MVPA was found to be inversely associated with BMI. Noteworthy, the majority of studies assessing the relationship between BMI and PA have employed subjective measures to assess the latter, namely self-reported questionnaires and surveys. Self-reported PA has been found to differ from more direct assessments, which questions the reliability of self-report measures [57]. The use of an accelerometer in a large sample in the present study therefore overcomes this methodological limitation.

In relation to the association between MVPA and temperament traits, an interesting link was found between MVPA and Novelty seeking and Harm avoidance scores. This is in line with other studies, whereby aspects of Novelty seeking, namely energetic attitude and exploratory behavior, have been found to be related to both motoric activity in animals [34] and weekly hours spent exercising in humans [58]. Whereas individuals with high scores in extraversion (corresponding to high Novelty seeking) have a more sociable and interactive lifestyle, and are thus more likely to be active [27], those with low levels of Novelty seeking tend to be passive, inhibited, and less dynamic, and are therefore expected to adopt a more sedentary lifestyle [26]. In contrast, the Harm avoidance temperament trait is associated with the inhibition of behaviors due to greater pessimistic worry or avoidance [59] and sensitivity to pain expectancy [60]. Individuals scoring higher in this trait are likely to be averse to engaging in MVPA, especially high contact and risky sports, as they avoid any activity in which they may be vulnerable to injury.

Consistent with prior animal [6] and human [20] studies, our results showed that AEA and OEA plasma concentrations but not 2-AG were positively associated with MVPA. Authors have proposed that this may be due to differences in the biosynthesis and degradation mechanisms [61]. Whereas AEA and OEA are N-acylethanolamides (NAEs) synthesized from the hydrolysis of *N*-arachidonoyl phosphatidylethanolamine (NAPE) and degraded by the FAAH, 2-AG is synthesized from other precursors (diacylglycerols) and enzymes (diacylglycerol lipases-a and b) and is primarily degraded by monoacylglycerol lipase (MAGL) [16]. Therefore, given that these eCBs have distinct metabolic pathways, a different interaction with MVPA has been proposed [6].

One discrepancy between our results and those of Heyman et al [57], is that we did not find an association between MVPA and PEA despite that PEA shares the same biosynthetic and degradation pathways of AEA and OEA. This could be because the eCB levels in this study were measured at the basal state, while Heyman et al [61] measured eCB levels after intense exercise. In support to our data, regarding the specific relationships between some NAEs and PA, Gasperi et al [62] found increased activity of FAAH in the lymphocytes of physically active subjects at resting condition. It must be noted that blood eCB levels represent the spillover from many sources and it is not possible to differentiate the tissue of origin. For instance, Caraceni et al [63] found that blood AEA, PEA and OEA levels were correlated with liver function, but eCB levels may vary differently in different tissues such as in the intestine or brain [12], [56], or in different depots of the same tissue such as in subcutaneous or visceral fat [14]. The skeletal muscle itself is altered in obesity, with increased expression of CB1 receptors and elevated levels of 2-AG without there being changes in AEA. It has been suggested that, in contrast to 2-AG, AEA and possibly OEA may have beneficial effects on glucose uptake and mitochondrial biogenesis in the muscle through the activation of other receptors such as peroxisome proliferators (PPARs) or the transient receptor potential vanilloid receptor 1 (TPRV1). In this regard, it has been proposed that PA could be a complementary approach for the treatment of obesity without the side effects of CB1 antagonists [19]. In addition to the PA peripheral effects, the potentiation of the eCB system after MVPA also has positive effects on cognitive functions, again linked to AEA [8], which could facilitate the implementation of both preventive and treatment programs for obesity.

Furthermore, in the current study the eCBs AEA and eCB-related compound OEA seem to act as contrasting mediators in the relationship between MVPA and BMI. The underlying mechanism between MVPA and the eCB system activation is not yet clear. It could be due to the increases in stress and glucocorticoid hormones (particularly cortisol) that occur with structured PA and seem to be implicated in the activation of eCB signaling [64]. Separately, eCBs have been found to be implicated in the regulation of appetite (as is the case of marijuana) via the activation of the reward system [65]. When energy homeostasis is challenged, such as in situations of food deprivation, an increase in endocannabinoid levels takes place [66]. The process is associated with a reinforced pleasure obtained from ingestion and from the rewarding properties of food [11], [67], which may lead to hyperphagia, overconsumption and consequentially weight gain [68]. Differently, the eCB-related compound OEA is a putative, peripheral satiety factor and anorexigen mediator, which promotes satiety and reduces weight gain by stimulating the vagal sensory nerves that in turn stimulate the brainstem and hypothalamus [13]. These findings are reflected in the mediation effect of AEA and OEA on the relationship between MVPA and BMI obtained in the current study. MVPA has an inverse direct effect on BMI, which can be attributed to energy expenditure, and a similar indirect relationship may also exist mediated by OEA. Yet, MVPA may also be associated with augmented BMI through the orexigen effect of AEA.

Finally, Novelty seeking and plasma AEA concentrations were both found to be positively linked to MVPA. Van Laere et al [31] evaluated CB1 receptor availability in temperament, finding greater global cerebral CB1 receptor availability to be inversely related to Novelty seeking. Novelty seeking may interact with the eCB system via the engagement in MVPA. Furthermore, the activation of this system with exercise appears to result in exercise-induced analgesia and may be responsible for the reported runner’s high, a transient and intense feeling of happiness, elation, and energy [20]. Our results support the concept that individuals who are high in impulsive traits may engage in PA to achieve a gratifying state, following a positive reinforcement conditioning, whereas more passive and less energetic individuals, such as those who present elevated Harm avoidance, present more sedentary behavior. Reduction in the BMI might be a consequent effect, but also may act as a maintaining factor of this vicious circle. Further research is needed to understand the mechanisms of these associations. For instance OEA is not a CB1 agonist and it is best known for acting as a fat sensor in the intestine, but in a recent report it has been suggested that OEA is also involved in the reward system by stimulating central dopamine activity [69] and may participate in the control of reward-related behaviors through a PPARα receptor-independent mechanism [70].

### Limitations

The study has a number of limitations that should be considered. First, the study focused of females. Future studies should also assess male participants who are likely to present distinct PA patterns. Second, the accelerometer was placed in the non-dominant wrist. Though this instrument permits a more accurate assessment of PA compared to subjective measures, PA entailing only lower-body movement may not be adequately captured. Future studies should place accelerometers to both the wrist and waist, as well as use self-reported questionnaires in order to obtain a more complete description of the activity patterns of participants. It must also be noted that in the current study plasma eCBs levels were assessed in the morning after an overnight fast, while MVPA was evaluated throughout the day. Although a link between time spent in MVPA and circulating eCBs was observed, a more controlled design should be developed in order to demonstrate the exact cause-effect mechanism. Finally, the cross-sectional nature of the study does not permit causality to be determined. Longitudinal studies should be conducted to evaluate how temperament in adolescence or young adulthood may predict the interaction between eCBs, temperament, and MVPA later in life, and to assess how lifestyle changes with increases in the time spent engaging in MVPA may be related to alterations in the eCB system and BMI.

Despite its limitations, this study has several important strengths, including the substantial sample size. Furthermore, path-analysis is used in this study as a case of structural equation modeling (SEM) with exploratory aims, with the advantage (compared to classical regression models) of allowing the inclusion of multiple relationships among a set of variables, including mediational associations. The results obtained constitute empirical evidence for the development of further theories about the role of MVPA, endocannabinoids and temperament on BMI.

In conclusion, the present study provides further understanding of the pathophysiological mechanism involved in PA and obesity by integrating previously described links between the eCB system, temperament, MVPA, and BMI, to generate a psychobiological model of the relationship between engagement in MVPA and BMI. It was shown that decreases in time spent in MVPA, rather than overall daytime PA, may be underlying the augmentation in obesity, and this may occur through the interaction with both psychological and biological factors. Important clinical conclusions may be drawn to confront the excess obesity among females. Future therapeutic approaches aiming at preventing obesity by reducing sedentary behavior and encouraging exercise, should consider both physiological and behavioral maintaining factors (e.g. attitude and motivation, behavioral tasks, environmental factors, locus of control), and temperament traits. It may be hypothesized that additional psychological interventions focusing on improving enthusiastic and inquiring attitudes, positive own reactions in front of novelty and new goals, might have a positive secondary influence on patients’ attitude towards MVPA.
